# Norcantharidin, Derivative of Cantharidin, for Cancer Stem Cells

**DOI:** 10.1155/2013/838651

**Published:** 2013-09-02

**Authors:** Chen-Hsi Hsieh, K. S. Clifford Chao, Hui-Fen Liao, Yu-Jen Chen

**Affiliations:** ^1^Division of Radiation Oncology, Department of Radiology, Far Eastern Memorial Hospital, New Taipei City 220, Taiwan; ^2^Department of Medicine, School of Medicine, National Yang-Ming University, Taipei 112, Taiwan; ^3^Institute of Traditional Medicine, School of Medicine, National Yang-Ming University, Taipei 112, Taiwan; ^4^Department of Radiation Oncology, Columbia University, NY 10032, USA; ^5^Department of Biochemical Science and Technology, National Chiayi University, Chiayi 600, Taiwan; ^6^Department of Radiation Oncology and Department of Medical Research, Mackay Memorial Hospital, Taipei 104, Taiwan

## Abstract

Cancer stem cells (CSCs) existing in human cancers have been demonstrated to be a major cause of cancer treatment resistance, invasion, metastasis, and relapse. Self-renewal pathways, Wnt/**β**-catenin, Sonic hedgehog (Shh), and the Notch signaling pathway play critical roles in developing CSCs and lead to angiogenesis, migration, invasion, and metastasis. Multidrug resistance (MDR) is an unfavorable factor causing the failure of treatments against cancer cells. The most important and thoroughly studied mechanism involved in MDR is the active efflux of chemotherapeutic agents through membrane drug transporters. There is growing evidence that Norcantharidin (NCTD), a water-soluble synthetic small molecule derivative of naturally occurring cantharidin from the medicinal insect blister beetle (*Mylabris phalerata* Pallas), is capable of chemoprevention and tumor inhibition. We summarize investigations into the modulation of self-renewal pathways and MDR in CSCs by NCTD. This review may aid in further investigation of using NCTD to develop more effective strategies for cancer treatment to reduce resistance and recurrence.

## 1. Introduction 

Cancer stem cells (CSCs) exist in many kinds of human cancers [1–5], and they are capable of continuous self-renewal and differentiation [6, 7]. In addition, CSCs may be responsible for tumor initiation, progression, metastasis, relapse, and resistance to chemotherapy or radiation therapy [8–11]. Several pathways, including Wnt/*β*-catenin, Hedgehog, and Notch, have been identified as playing pivotal roles in CSC self-renewal [12–14], leading to relapse and multidrug resistance [15]. 

After developing resistance to a single drug or a class of drugs, cancer cells show cross-resistance to other functionally and structurally unrelated drugs, causing the failure of treatments against cancer cells [16]. This phenomenon is known as multidrug resistance (MDR). MDR has an unfavorable effect on successful outcomes of chemotherapy against cancer [17]. MDR can reduce intracellular drug accumulation by the active efflux of chemotherapeutic agents to modulate the expression of target genes controlling the cell cycle, cell adhesion, signal transduction, vascularization, and apoptosis. 

**Figure 1:**
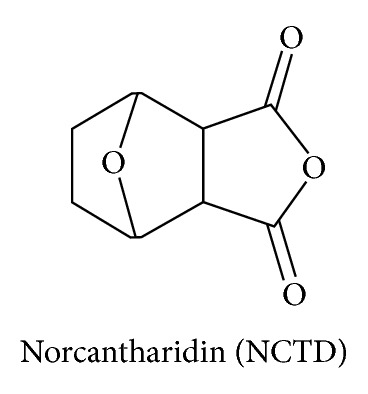
Chemical structure of norcantharidin (NCTD).

Norcantharidin (NCTD, exo-7-oxabicylo-[2.2.1] heptane-2,3-dicarboxylic anhydride), a water-soluble synthetic small molecule, is a demethylated analog of cantharidin (CTD, 7-oxabicyclo-[2.2.1] heptane-2,3-dicarboxylic acid) [18]. The molar weight, complexity, and heavy atom count for NCTD are 168.15 g/mol, 246, and 12, respectively (Figure 1). CTD is a naturally occurring compound isolated from the medicinal insect blister beetle (*Mylabris phalerata *Pallas) [18]. The most important of the medicinal uses of CTD is its anticancer activities [18]. It is capable of inducing p53-dependent apoptosis and double-strand breakage of DNA in cancer cells [18–22]. CTD treatment could cause granulocytosis *in vivo* but not granulocytopenia induced by most chemotherapeutics [18]. This unique bioactivity renders CTD a promising lead compound for chemical modification to develop cancer therapeutics. However, the application of CTD is limited due to its toxicity to gastrointestinal and urinary tracts [23]. NCTD causes fewer nephrotoxic and inflammatory side effects than CTD [18, 23], and like CTD has been demonstrated as a potential agent against certain cancers [24]. The cytotoxic and antitumor activities of NCTD are multifarious: it can cause apoptosis, inhibition of angiogenesis, and metastasis for many cell lines, and it can affect multiple pathways controlling cell proliferation [25–27]. Moreover, NCTD was found able to inhibit P-glycoprotein (P-gp) [28] and overcome MDR [29]. 

NCTD decreased hepatic leukemia factor (HLF) protein levels, a gene implicated in hematopoietic stem cell (HSC) regulation, and induced apoptosis in the acute myeloid leukemia (AML) cell line MV4-11 by modulating the expression of several molecules that govern survival pathways, including HLF, SLUG, NFIL3, and c-myc, thereby inducing p53 and the mitochondrial caspase cascade that explores the ability of NCTD to target stem cells [30]. NCTD encapsulated liposomes modified with a novel murine anti-human CD19 monoclonal antibody 2E8 (2E8-NCTD-liposomes) could specifically target the B-lineage leukemia stem cells (B-LSCs) and their progeny *in vitro *[31]. Their results have shown that the internalization of 2E8-NCTD-liposomes into the cells and the subsequent release of NCTD into the cytoplasm to induce the apoptosis of B cells were responsible for specific cytotoxicity to the cells, using confocal microscopy and multiparameter flow cytometry analyses. In addition, immunoliposomes were able to induce the apoptosis of B-LSCs via downregulating the HLF and upregulating the NFIL3 (nuclear factor, IL3 regulated) expressions at the mRNA level, proved by real-time RT-PCR [31].

Besides inhibiting cancer cells, NCTD also affects normal cells. NCTD inhibits peripheral blood mononuclear cell (PBMC) proliferation with a 50% inhibitory concentration (IC (50)) 42.1 ± 2.3 microM without direct cytotoxicity or the arrest of cell-cycle progression in the cells [37]. NCTD modulates the differentiation and maturation of human myeloid DCs and causes deviation of standard DC differentiation toward a tolerogenic phenotype through calcineurin phosphatase inhibition and, thus, has potential for development as an immunosuppressant for transplant rejection [47]. NCTD is protective against renal tubulointerstitial fibrosis both *in vivo* and *in vitro *[48, 49]. Epithelial-mesenchymal transition (EMT) contributes to the progression of renal tubulointerstitial fibrosis. NCTD antagonizes tubular EMT by inhibiting the TGF-beta1/Smad pathway, which suggests that NCTD may play a critical role in preserving the normal epithelial phenotype and modulating tubular EMT [48]. On high glucose-induced extracellular matrix (ECM) and TGF-beta1 in human kidney proximal tubular epithelial (HK-2) cells, the antifibrogenic effect of NCTD on tubular interstitium in diabetic nephropathy (DN) is independent of calcineurin (CaN)/Nuclear Factor of Activated T-cell (NFAT) pathway inhibition [49]. However, Yan et al. [50] also noted that NCTD has no effect on inactive lymphocytes but selectively acts on activated lymphocytes. These data support the multiple abilities of NCTD to influence cancer cells, CSCs, or normal cells.

In this paper we review the current understanding of NCTD, which has cancer treatment potential, with a focus on overcoming MDR and CSC self-renewal characteristics (Table 1).

## 2. Self-Renewal Pathways of Cancer Stem Cells 

### 2.1. Wnt/*β*-Catenin Pathway

The Wnt/*β*-catenin pathway modulates cell proliferation, migration, apoptosis, differentiation, and stem cell self-renewal [51–53]. *β*-Catenin participates in two distinct functions in the cell. Membrane-localized *β*-catenin is a protein adhesive that with E-cadherin maintains cell-cell adhesion [54]. Cytoplasmic accumulation of *β*-catenin cooperates with the transcription factors T cell factor/lymphoid enhancer factor (TCF/LEF) as a transcription activator, which eventually leads to activation of Wnt target genes such as *c-Jun*, *c-Myc*, *fibronectin*, and *cyclin D1 *[55–60].

**Figure 2:**
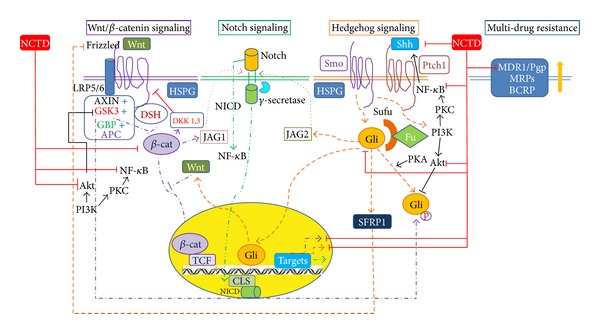
Model of crosstalk between hedgehog signaling, Wnt/*β*-catenin signaling, notch signaling, and phosphoinositide 3 (PI3)-kinase/Akt pathway and targeting by norcantharidin (NCTD). *β*-cat: *β*-catenin; BCRP: breast cancer resistance protein; Fu: fused; HSPG: Gli: glioma-associated oncogene family zinc finger; GSK-3: glycogen synthase kinase 3; Heparin-sulfated forms of proteoglycans; NICD: intracellular domain of Notch; JAG: Protein jagged; LRP: Low-density lipoprotein receptor-related protein; MDR: multidrug resistance; MRPs: multidrug resistance proteins; NCTD: norcantharidin; P-gp: P-glycoprotein; PI3K: phosphoinositide 3-kinase; PKA: protein kinase A; Ptch: patched; SFRPs: secreted frizzled receptor proteins; Shh: sonic hedgehog; Smo: smoothened; Sufu: suppressor of fu.

Increasing evidence supports the ability of NCTD to inhibit the Wnt/*β*-catenin pathway. Cimmino et al. [32] reported that NCTD could impair the growth of medulloblastoma cells and promoted the loss of beta-catenin activation. Additionally, the Wnt/*β*-catenin signaling pathway contributes to refractory and relapsed leukemia. Chuang et al. [33] also confirm NCTD as an inhibitor for the Wnt/*β*-catenin pathway. They note that NCTD (50 microM) inhibits the proliferation of Jurkat cells with dominant beta-catenin signaling by 64% in a concentration-dependent manner. In CT26 colorectal adenocarcinoma cells, NCTD decreases the adhesive ability of CT26 cells and shows a downregulation of several cadherin-catenin adhesion molecules *in vitro.* It could reduce both the pulmonary metastatic capacity of CT26 cells and prolong the survival time of the tumor-bearing mice [34] (Figure 2).

NCTD also inhibits the activation of Wnt target genes such as *c-Jun* and *cyclin D1. *In human gallbladder carcinoma xenografted tumors, an NCTD-treated group decreased the expression of cyclin-D1, Bcl-2, and survivin proteins/mRNAs significantly [35]. Similar results were noted in human gallbladder carcinoma GBC-SD cells *in vitro *[36]. NCTD inhibits the growth of GBC-SD cells by increasing the rate of cell apoptosis and decreasing the expression of the proliferation-related genes, such as cyclin-D1 or the apoptosis-related genes [36]. NCTD also arrests the cell-cycle progression from the G1 transition to the S phase through declining cyclin D3, E, A, and B transcripts and stops protein production in phytohemagglutinin (PHA-) treated peripheral blood mononuclear cells (PBMC) [37]. 

### 2.2. Hedgehog Pathway

The Hedgehog (Hh) signaling pathway plays a major role as regulator of cell differentiation, tissue polarity and cell proliferation [61, 62]. There are three secreted proteins belonging to the Hh family, including Sonic Hedgehog (Shh), Desert hedgehog, and indian hedgehog. In the absence of hedgehog ligands, the transmembrane receptor Patched (Ptch) blocks the Smoothened (Smo) function [63–65]. If secreted hedgehog ligands bind to Ptch1, then Smo is reversed to activate the Shh signaling pathway, resulting in the translocation of the transcription factor Gli (glioma-associated oncogene family zinc finger) family into the nucleus to modulate the expression of target genes, such as *cyclin D*, *cyclin E*, *Myc*, and elements of the EGF pathway, which control the cell cycle, cell adhesion, signal transduction, vascularization, and apoptosis [63–67]. Hh plays a central role in the control of proliferation and differentiation of both embryonic stem cells and adult stem cells; the aberrant activation of Hh signaling could lead to the generation of CSCs and the development of cancer [68] or cancer angiogenesis, metastasis, and invasion [69]. 

The plasma VEGF levels of tumor-bearing mice, migration, and capillary-like tube formation of HUVECs are suppressed by NCTD with potential antimetastasis and antiangiogenesis [38]. Chen et al. [39] demonstrated that the Shh expression for various cell lines of breast cancer is suppressed by NCTD and the nuclear translocation of Gli-1is inhibited as well. NCTD inhibits metastasis in CT26 cells by the downexpression of matrix metalloproteinase-9 (MMP-9) activity and of several cadherin-catenin adhesion molecules [34] through inhibiting the transcriptional activity of Sp1 [40] (Figure 2).

### 2.3. The Crosstalk between Hedgehog Signaling, Wnt/*β*-Catenin, Notch Signaling, and Phosphoinositide 3 (PI3)-Kinase/Akt Pathway

There are crosstalks between hedgehog signaling, Wnt/*β*-catenin, notch signaling, and the phosphoinositide 3-kinase (PI3-kinase)/Akt pathway. These signaling molecules are activated by G-protein-coupled receptors, such as Frizzled or Smo [70, 71]. The pathways prevent phosphorylation-dependent proteolysis of key effectors, cubitus interruptus, or *β*-catenin [72]. The study notes that activation of Gli stimulates the transcription of Wnt ligands [64]. The molecule in Wnt signaling, glycogen synthase kinase (GSK)-3*β*, regulates the molecules involved in Hh signaling [73], but the pathological response to oncogenic Hh signaling is also dependent on canonical Wnt/*β*3-catenin signaling [74]. Taken together, it is apparent that crosstalk between Wnt and Hh signaling is evident. 

The PI3-Kinase/Akt pathway links to the Wnt/*β*-catenin pathway. The PI3-Kinase/Akt pathway acts as a survival signal and plays a key role in the regulation of apoptotic events. The PI3-Kinase/Akt pathway is important in regulating the mammary stem/progenitor cells by promoting *β*-catenin downstream through phosphorylation of GSK-3*β*. Activated Akt was shown to be able to phosphorylate Ser9 on GSK-3*β*, which may decrease the activity of GSK-3*β*, thereby stabilizing *β*-catenin [75, 76]. Akt can exert its antiapoptotic effects in several different ways, such as negatively regulating proapoptotic factors and stimulating the nuclear factor-kappaB (NF-*κ*B) survival pathway [77]. NF-*κ*B can promote tumorigenesis and is linked to cell invasion and metastasis. The suppression of NF-*κ*B activation is effective in the prevention and treatment of cancer [78]. 

More reports have demonstrated the direct or synergistic role of PI3-kinase/Akt activation in mediating the biological effects of hedgehog signaling [79–82]. Genetic studies in mice reveal that the insulin-like growth factor (IGF)-PI3-kinase/Akt pathway provides a synergistic signal for Shh in tumor formation [83, 84]. Akt positively regulates Shh signaling by controlling protein kinase A-(PKA-) mediated Gli inactivation [79]. Shh induces capillary morphogenesis of endothelial cells through activation of c-Fes/PI3-kinase pathways [82] noted in the angiogenic study of bone-marrow-derived endothelial progenitor cells (BM-EPC) [81]. Shh signaling could promote the metastasis of gastric cancer cells through the activation of the PI3K/Akt pathway, which may lead to epithelial mesenchymal transition and MMP-9 activation [85]. Shh may protect the astrocytes from oxidative stress by activating the PI3-Kinase/AKT pathway [86].

Likewise, the Shh pathway is linked to transcription factor NF-*κ*B signaling. It has been suggested that overexpression of Shh is activated by NF-*κ*B in pancreatic cancer and pancreatic cancer cell proliferation is accelerated by NF-*κ*B in part through Shh overexpression [87]. Kasperczyk et al. further characterized Shh as a novel NF-*κ*B target gene and mapped a minimal NF-*κ*B consensus site to position +139 of the Shh promoter [88]. 

Notch signaling is known to control cell proliferation and apoptosis to modulate the development of many organs [89]. A number of recent studies have demonstrated that Notch-activated genes and pathways can drive tumor growth through the expansion of CSCs [89–94]. Notch 1, a transmembrane receptor, has been reported to crosstalk with the NF-*κ*B pathway in diverse cellular situations [95–97]. Specifically, Notch-1 is necessary for the expression of several NF-*κ*B subunits [96, 98], and it stimulates NF-*κ*B promoter activity [96].

Activation of PI3K/Akt and NF-*κ*B increases the migration of cancer cell lines such as human lung cancer A549 cells [99] and human breast cancer MDA-MB-231 cells [100]. NCTD dose-dependently suppresses the phosphorylation of Akt and NF-*κ*B expression in human breast cancer MDA-MB-231 cells [41]. Moreover, NCTD reduces the human lung cancer A549 cell migration by more than 65% at low concentrations (0.2–0.8 *μ*g/mL) without affecting cell viability [42]. Activation of extracellular signal-regulated kinase (ERK), c-Jun NH2-terminal kinase (JNK) and the modulation of downstream transcription factor NF-kB are involved in NCTD-induced apoptosis for human hepatoma HepG2 cells [43]. Similarly, NTCD is effective as a c-Jun N-terminal kinase inhibitor, SP600125, for breast cancer cells (HS-578T) [26]. NCTD can inhibit ERK1/2 phosphorylation effectively, by reducing NF-*κ*B DNA-binding activities, leading to matrix metalloproteinases (MMP)-9 downregulation and u-plasminogen activator (PA) expression to reduce the invasion of hepatocellular carcinoma (Huh7) cells [44]. It suggests that NCTD, not solely due to viability inhibition, may inhibit the PI3-K/Akt pathway to contribute activity against CSCs (Figure 2).

## 3. Multidrug Resistance (MDR)

Multidrug resistance (MDR) is an unfavorable factor causing the failure of treatments against cancer cells [16]. It occurs when cancer cells acquire simultaneous resistance to various kinds of chemotherapeutic agents with no structural or functional similarities [101]. Although many mechanisms of MDR in cancer cells have been studied, the most important and thoroughly studied mechanism involves the reduction in intracellular drug accumulation by the active efflux of chemotherapeutic agents through membrane drug transporters. These ATP-binding cassette (ABC) proteins include p-glycoprotein (P-gp, MDR1, and ABCB1) [17, 102, 103], the multidrug resistance protein 1 (MRP1) [104, 105], lung resistance protein (LRP) [106, 107], and breast cancer resistance protein (BCRP, ABCG2) [108–111]. The P-gp acts as a drug efflux pump to extrude a wide range of different chemotherapeutic drugs out of MDR cancer cells [102].

NCTD is found to inhibit the P-gp [28] and the multidrug resistance-associated protein 2 (MRAP 2) to significantly enhance the uptake amount of nanoparticles with lactosyl-norcantharidin in a heterogeneous human epithelial colorectal adenocarcinoma cells monolayer model [45]. NCTD irreversibly reduced the clonogenic efficiency of parental and drug-resistant K562 sublines, with drug-resistant sublines showing greater susceptibility to NCTD than parental cells. The data suggest that NCTD may be suitable in the treatment of drug-resistant leukemia [29]. Similarly, apoptosis of oral cancer cells with resistance to multiple chemotherapeutic agents can be induced by NCTD [46]. In a study of doxorubicin-(DOX-) resistant human breast cancer MCF-7R cells, NCTD increased the intracellular accumulation of DOX in MCF-7R cells and suppressed the upregulation of the MDR-1 mRNA, P-gp and BCRP protein expression but not MRP-1 [39]. Collectively, it is apparent that NCTD might be the substrate of P-gp and might overcome multidrug resistance in cancer cells.

## 4. Prospect of Using Norcantharidin against Cancer Stem Cells

The experimental demonstration of CSCs in several human tumors in recent years promises a new cellular target for anticancer drug discovery [1, 4, 5, 112–114]. Among various agents that target self-renewal pathways, small molecules that target the hedgehog pathways are in early clinical studies and have shown promising results [115, 116]. The Smo antagonist cyclopamine was shown to lead to the rapid regression of basal cell carcinoma in patients [117]. In addition, an oral small molecule inhibitor of Smo, GDC-0449, has shown limited toxicity and partial responses in advanced basal cell carcinoma tumors in a Phase I clinical trial, and it is advancing to Phase II trials for metastatic colorectal cancer and other advanced epithelial tumors [118]. Given that the expression of SHH in various breast cancer cell clines and the nuclear translocation of Gli-1 are suppressed by NCTD [39], it may imply that NCTD can be used to target renewal signaling against CSCs.

The Wnt/*β*-catenin pathway initiates a signaling cascade critical in both normal development and the initiation and progression of cancer [119–121]. Various *β*-catenin/TCF inhibitors, most of them belonging to low molecular-weight inhibitors, downregulate the expression of *β*-catenin/TCF-responsive genes and disrupt the interaction of element-binding protein (CBP) with *β*-catenin [122] or disrupt *β*-catenin/TCF complexes directly [123]. Similarly, NCTD can promote the loss of *β*-catenin activation [32] and inhibit the proliferation of Jurkat cells with dominant *β*-catenin signaling [33]. These data suggest that NCTD has significant therapeutic potential for the treatment of cancer with activated Wnt/*β*-catenin pathways.

Epithelial cell adhesion molecule (EPCAM) is highly expressed in numerous solid tumors, and it has recently been shown to be expressed on tumor-initiating cells from breast, prostate, colon, and pancreatic cancer [3, 113, 124]. There are several antibodies against cell surface markers of tumor-initiating cells in clinical studies [125, 126]. However, EPCAM-specific mAbs have shown a limited efficacy in clinical trials [125]. These data suggest that an immune response stimulated by these mAbs by itself might not be effective in killing EPCAM overexpressing tumor cells in clinical settings. To overcome the limitations of the naked antibodies, catumaxomab, a trifunctional antibody against EPCAM and CD3, brings cancer cells into proximity with the immune system cells that can destroy them [127]. NCTD is not only cytotoxic for cancer cells but also plays a role in modulating the development of dendritic cells to prolong skin allograft survival [47]. With the multiple roles of NCTD, it needs to be determined whether NCTD can modulate immune tolerance or antibody-dependent cellular cytotoxicity (ADCC) to kill EPCAM overexpressing tumor cells in a microenvironment.

Radiation sensitization is one of the important directions to develop anticancer agents for radiotherapy or chemoradiation therapy. Shh signaling has been discovered as a mechanism rendering cancer cells resistant to chemoradiation. In clinical practice, the expression of Ptch or Gli-1 has been significantly associated with a poor prognosis for oral cavity cancer patients [128]. Cancer cells at the G2/M phase are known to be sensitive to radiation [129, 130]. NCTD could suppress the expression of Shh and Gli-1for various cell breast cancer lines [39]. NCTD can significantly increase the proportion of cells in G2/M phase and decrease the proportion of cells in S phase for CT26 colorectal adenocarcinoma cells [27], gallbladder carcinoma GBC-SD cells [36], and human breast cancer MDA-MB-231 cell lines [41]. Taken together, NCTD may play a dual role as radiosensitizer and CSC toxic agent. 

Safety issues are worth noting in efforts to develop chemotherapy-enhancing or radiation sensitization agents aimed at eliminating CSCs. Research efforts should be oriented toward avoiding, or at least minimizing, the inhibition of crucial mechanisms for normal stem cell maintenance. 

NCTD has pharmacological potential in the treatment of CSCs. The beneficial effects of NCTD include the modulation of CSC self-renewal pathways, overcoming multidrug resistance and as radiation sensitizer. Although the mechanisms are not clearly addressed in the reviewed publications, the results indicate that further evaluation of NCTD is warranted. In particular, the mechanisms of action by which NCTD modulates CSC characteristics should be clarified. Preclinical studies to pave the way for clinical trials may eventually enable scientists to discover more effective strategies for cancer treatment to reduce resistance and recurrence and, eventually, to improve survival of cancer patients.

## Figures and Tables

**Table 1:** Summary of norcantharidin (NCTD) against human cancer stem cells (CSCs) and cancer cells.

Target class	Target demonstrated	Effects and mechanisms	Comments	References
Stem cells	*Hepatic leukemia factor (HLF) protein levels, a gene implicated in hematopoietic stem cell (HSCs). *Acute myeloid leukemia (AML) cell line MV4-11.	*NCTD decreased HLF protein levels, a gene implicated in hematopoietic stem cell (HSCs) regulation. *NCTD induced apoptosis in the AML cell line MV4-11 by modulating the expression of molecules that govern survival pathway, including HLF, SLUG, NFIL3, and c-myc, inducing p53 and the mitochondrial caspase cascade	Explores the ability of NCTD to target stem cells.	[30]
	B-lineage leukemia stem cells	2E8-NCTD-liposomes into the cells and NCTD into the cytoplasm to induce the apoptosis of B cells.		[31]

Wnt/β-catenin pathway	DAOY and UW228 medulloblastoma cells	NCTD crosses the blood-brain barrier, inhibits the growth of medulloblastoma cells, and impairs the Wnt-beta-catenin signaling.	NCTD impairs the growth of medulloblastoma cells through inhibition of Wnt-beta-catenin signaling.	[32]
	HEK 293-TOP and Jurkat-TOP stable clones	NCTD inhibits proliferation of Jurkat cells, which are the dominant beta-catenin signaling cells, in a concentration-dependent manner.	NCTD is an inhibitor of Wnt/beta-catenin signaling.	[33]
	CT26 colorectal adenocarcinoma cells	NCTD downregulates expression of desmoglein, N-cadherin, and alpha- and beta-catenin, while there were no obvious changes in E-cadherin and gamma-catenin in colorectal cancer CT26 cells.	NCTD is effective in blocking both tumor invasion and metastasis.	[34]

The activation of Wnt target genes: *c-Jun* and *cyclin D1 *	Human gallbladder carcinoma GBC-SD cells xenografted tumors	NCTD inhibits the growth of the xenografted tumors in a dose- and time-dependent manner and decreases the expression of cyclin-D1, Bcl-2, and survivin proteins/mRNAs significantly.	NCTD inhibits the growth of xenografted tumors of human gallbladder carcinoma in nude mice by inducing apoptosis and blocking the activation of Wnt target genes, cyclin-D1.	[35]
	Human gallbladder carcinoma GBC-SD cells	NCTD inhibits cell proliferation, arrest of the cell cycle, blockage of DNA synthesis, induction of cell apoptosis and influence on expression of the proliferation-related genes PCNA, Ki-67, cyclin-D1 and p27, and the apoptosis-related genes Bcl-2, Bax, and survivin in human gallbladder carcinoma GBC-SD cells.	NCTD inhibits the growth of human gallbladder carcinoma GBC-SD cells *in vitro* and decreases the expression of cyclin-D1 in human gallbladder carcinoma GBC-SD cells.	[36]
	Phytohemagglutinin-(PHA-) treated peripheral blood mononuclear cells (PBMC)	NCTD reduces the cyclin D3, E, A, and B transcripts and protein production in PBMC.	NCTD suppresses the proliferation of PBMC activated by PHA through inhibition of cyclins and IL-2 production.	[37]

Hedgehog pathway	Human umbilical vein endothelial cells (HUVECs)	NCTD inhibits migration and capillary-like tube formation of HUVECs. The antiangiogenic effect of NCTD is accompanied by anoikis, downregulation of integrin beta1 and breakdown of vimentin.	NCTD inhibited the release of proangiogenic factors from HUVECs.	[38]
	Human breast cancer MCF-7 cells, MDA-MB-231 and BT-474 cells	NCTD suppresses the upregulation of Shh expression and nuclear translocation of Gli-1, a hallmark of Shh signaling activation in the resistant clone.	NCTD overcomes multidrug resistance through inhibiting Shh signaling and expression of its downstream mdr-1/P-gp expression in human breast cancer cells.	[39]
	CT26 colorectal adenocarcinoma cells	NCTD downregulates matrix metalloproteinase-9 (MMP-9) expression by inhibiting Sp1 transcriptional activity and suppresses the activation of several cadherin-catenin adhesion molecules of desmoglein, N-cadherin, and alpha- and beta-catenin in colorectal cancer CT26 cells.	NCTD inhibits metastasis in CT26 cells by the downexpression of MMP-9 activity through inhibiting transcriptional activity of Sp1.	[34, 40]

The crosstalk between Hedgehog signaling, Wnt/*β*-catenin, notch signaling, and phosphoinositide 3 (PI3)-kinase/Akt pathway	Human breast cancer MDA-MB-231 cells	NCTD induces apoptosis and cell cycle arrest as well as reduction of Bcl-2/Bax ratio that may be the important mechanisms of action of NCTD suppressing the growth of MDA-MB-231 cells, which are associated with inhibition of the Akt and NF-kappa B signaling pathway.	NCTD dose-dependently suppresses the phosphorylation of Akt and NF-*κ*B expression in human breast cancer MDA-MB-231 cells.	[41]
	Human nonsmall cell lung cancer A549 cell lines	NCTD reduces the human lung cancer A549 cell by more than 65% at low concentrations (0.2–0.8 *µ*g/mL) without affecting the cell viability.	NCTD reduces the human lung cancer A549 cell migration rate.	[42]
	Human hepatoma HepG2 cells	Activation of extracellular signal-regulated kinase (ERK), c-Jun NH2-terminal kinase (JNK) and modulation of downstream transcription factor NF-*κ*B are involved in NCTD-induced apoptosis for human hepatoma HepG2 cells	NCTD activates NF-kappa B through Ikappa B kinase (IKK)-dependent phosphorylation pathway for HepG2 cells.	[43]
	Human breast cancer cells (HS-578T)	NTCD activates mitogen-activated protein kinases (MAPKs) family member proteins, extracellular signal-regulated kinase (ERK), p38(MAPK), and c-Jun N-terminal kinase (JNK) for breast cancer cells (HS-578T).	NTCD may be an effective anti-cancer drug against breast cancer through MAPK and signal transducers and activators of transcription (STATs) pathways.	[26]
	Hepatocellular carcinoma (Huh7) cells	NCTD can inhibit ERK1/2 phosphorylation effectively, by reducing NF-*κ*B DNA-binding activities, leading to matrix metalloproteinases (MMP)-9 downregulation and u-plasminogen activator (PA) expression to reduce the invasion of hepatocellular carcinoma (Huh7) cells.	NCTD inhibits MMP-9 and u-PA expression through the phosphorylation of ERK1/2 and NF-kappaB signaling pathway for Huh7 cells.	[44]

Multi-drug resistance (MDR)	The intestinal absorption mechanisms	The absorption rate constants (Ka) of NCTD at different segments were found to be duodenum > jejunum > ileum > colon. The transport of NCTD is found to be inhibited by P-glycoprotein (P-gp) inhibitor.	NCTD might be the substrate of P-gp.	[28]
	Human epithelial colorectal adenocarcinoma cells (Caco-2) cell	The inhibitor of P-gp and the multidrug resistance-associated protein 2 (MRAP 2) significantly enhances the uptake amount of lactosyl-norcantharidin (Lac-NCTD).	Lac-NCTD-nanoparticles (NPs) could be the substrate of P-gp and the MRAP 2 for Caco-2 cells.	[45]
	Human myeloid leukemia cells K562	NCTD irreversibly reduced the clonogenic efficiency of drug-resistant K562 sublines, showing greater susceptibility to NCTD.	NCTD may be suitable in the treatment of drug-resistant leukemia.	[29]
	Human oral cancer cell lines SAS (p53 wild-type phenotype) and Ca9-22 (p53 mutant)	Oral cancer cells with mutant p53 or elevated Bcl-XL levels showed resistance to multiple chemotherapeutic agents. NCTD downregulates the expression of Bcl-2 in Ca9-22 and Bcl-XL in SAS.	NCTD may overcome the chemoresistance of oral cancer cells with mutant p53 or elevated Bcl-XL levels.	[46]
	Doxorubicin-(DOX-) resistant human breast cancer MCF-7R cells	NCTD increased the intracellular accumulation of DOX in MCF-7R cells and suppressed the upregulation of the MDR-1 mRNA, P-gp, and BCRP protein expression.	NCTD may overcome multidrug resistance through inhibiting Shh signaling and expression of its downstream mdr-1/P-gp in human breast cancer cells.	[39]

*The hepatic leukemia factor (HLF) is one of the most consistently overexpressed genes in the leukemic stem cells (LSCs) compartment.
